# Deep learning segmentation of non-perfusion area from color fundus images and AI-generated fluorescein angiography

**DOI:** 10.1038/s41598-024-61561-x

**Published:** 2024-05-11

**Authors:** Kanato Masayoshi, Yusaku Katada, Nobuhiro Ozawa, Mari Ibuki, Kazuno Negishi, Toshihide Kurihara

**Affiliations:** 1 Laboratory of Photobiology, Keio University School of Medicine, 35 Shinanomachi, Shinjuku-Ku, Tokyo Japan; 2 Department of Ophthalmology, Keio University School of Medicine, Shinanomachi, Shinjuku-Ku, Tokyo Japan

**Keywords:** Deep Learning, Generative Artificial Intelligence, Retinal Vein Occlusion, Fluorescein Angiography, Retinal diseases, Translational research, Image processing, Machine learning

## Abstract

The non-perfusion area (NPA) of the retina is an important indicator in the visual prognosis of patients with branch retinal vein occlusion (BRVO). However, the current evaluation method of NPA, fluorescein angiography (FA), is invasive and burdensome. In this study, we examined the use of deep learning models for detecting NPA in color fundus images, bypassing the need for FA, and we also investigated the utility of synthetic FA generated from color fundus images. The models were evaluated using the Dice score and Monte Carlo dropout uncertainty. We retrospectively collected 403 sets of color fundus and FA images from 319 BRVO patients. We trained three deep learning models on FA, color fundus images, and synthetic FA. As a result, though the FA model achieved the highest score, the other two models also performed comparably. We found no statistical significance in median Dice scores between the models. However, the color fundus model showed significantly higher uncertainty than the other models (p < 0.05). In conclusion, deep learning models can detect NPAs from color fundus images with reasonable accuracy, though with somewhat less prediction stability. Synthetic FA stabilizes the prediction and reduces misleading uncertainty estimates by enhancing image quality.

## Introduction

Branch retinal vein occlusion (BRVO) is a vision-threatening disease caused by blocked retinal veins. In the assessment of BRVO, non-perfusion areas (NPA) on the retina are a key indicator for prognosis and treatment. To evaluate NPAs, a normal color fundus image is insufficient. Instead, ophthalmologists primarily rely on fluorescein angiography (FA), which provides rich information about vascular leakage, capillary vessels, and microaneurysms by using a contrast agent (fluorescein)^1^. However, the intravenous infusion of fluorescein is invasive and requires significant time and human resources^2^. While optical coherence tomography angiography (OCTA), a novel imaging method for the retina, can be a noninvasive alternative to FA, it requires an expensive device and therefore has limited accessibility^3–5^.

To offer a safer and more affordable diagnostic method of BRVO, two AI approaches have been previously proposed: (1) segmentation AI that can predict NPA from only color fundus^6–9^ and (2) generative adversarial network (GAN) models that can translate color fundus images into synthetic FA-like images^10–14^. These approaches have the potential to allow BRVO patients to avoid costly or invasive examinations.

Nonetheless, there were knowledge gaps that needed to be filled. First, there was an insufficient comparison between AI models using the gold standard method (FA) and ones using color fundus images. Although it is reported that AI could predict NPAs using only color fundus images, that is insufficient to determine whether color fundus images can replace FA in BRVO diagnosis because FA models might perform better enough to accept the cost and potential adverse effects of FA. Second, the diagnostic utility of synthetic FA was unclear. Though the GAN model researchers have shown potential benefits such as enhancing the retinal vessels that are hardly visible in color fundus images, the clinical benefits of synthetic FA should be clearly demonstrated.

**Figure 1:**
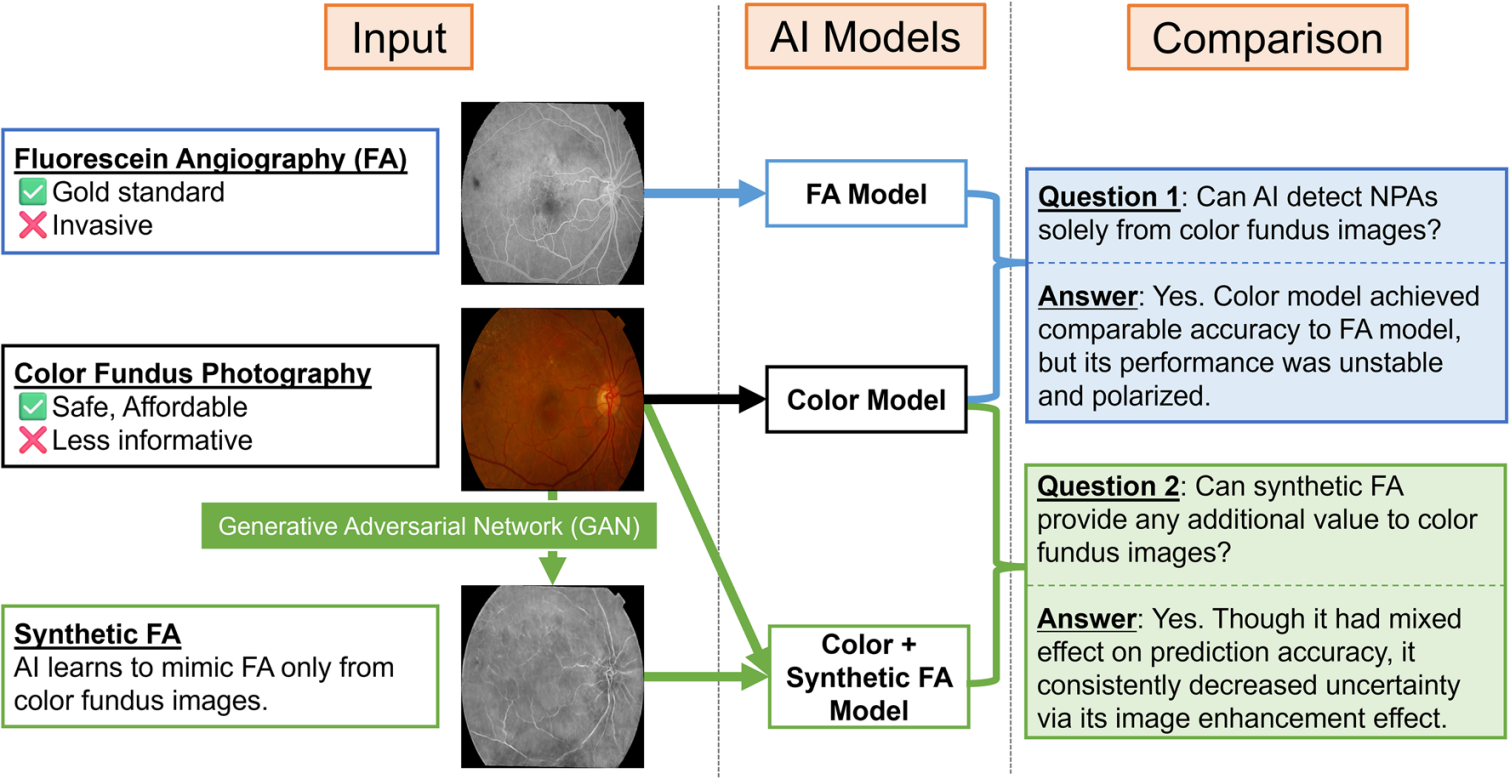
Model training and research questions. The figure shows the abstract of the present research. We trained three deep learning models on different input sources. By comparing these models, we answered two research questions regarding the utility of color fundus and synthetic FA images.

To address these knowledge gaps, we quantitatively compared three deep learning models, each trained on different types of images (Fig. 1). FA model was trained on FA images, which is expected to perform the best as it uses the gold standard modality. The color fundus model was trained on color fundus images. The color fundus + synthetic FA model was trained on color fundus images and synthetic FA images generated from color fundus images. The last two models do not require real FA images hence less invasive and costly, but the performance might deteriorate compared to the FA model. Through these experiments, the present study aimed to address the following questions:

- (1) Can deep learning models reliably detect NPAs using only color fundus images with the same accuracy as models using FA?
- (2) Can synthetic FA provide additional value over color fundus images in NPA prediction?

## Results

### Dataset

**Figure 2:**
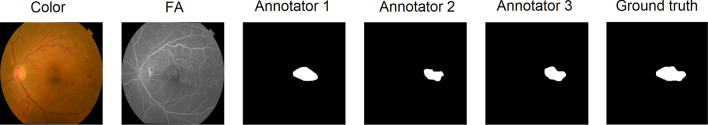
Example of preprocessed and annotated images. Three licensed ophthalmologists aligned the color fundus and FA images, and then they independently annotated the NPA. We defined the ground truth as the union set of the three annotations. Generated FA images are not shown here since they were generated from the color fundus images and were not raw data.

We retrospectively collected 403 pairs of color fundus and FA images from 319 BRVO patients at Keio University Hospital, Tokyo, Japan. Table 1 shows the demographic characteristics of the dataset. Three ophthalmologists created NPA annotation (Fig. 2) and the inter-annotator agreement is shown in Table 2.

**Table 1:** Demographic characteristics of the dataset (mean ± 95% confidence interval (CI).

Demographics (n = 403)	
Age	65.7 ± 0.6
20–50	44 (10.9%)
50–80	313 (77.7%)
80–100	46 (11.4%)
Systolic blood pressure (mmHg)	130.1 ± 1.0
Diastolic blood pressure (mmHg)	76.6 ± 0.7
Sex	
Male	212 (52.6%)
Female	191 (47.4%)
NPA ratio (%)	34
(No NPA)	80 (19.9%)
0–30%	124 (30.8%)
30–80%	186 (46.2%)
80–100%	13 (3.2%)
Ethnicity	
Japanese	403 (100%)

The NPA ratio is the ratio of NPA pixels to all fundus pixels in the image.

**Table 2:** Inter-annotator agreement measured by Dice score (%).

Dice score (%)	A	B	C	Ground truth
A	100	87.7 [52.6, 97.4]	94.9 [77.5, 99.7]	99.5 [97.8, 100]
B	–	100	96.4 [82.8, 99.9]	88.9 [56.7, 97.9]
C	–	–	100	97.9 [87.3, 100]

Median Dice scores are shown with the interquartile ranges (IQR) in the brackets.

### Synthetic FA generation

The similarity metrics of the synthetic FA and color fundus are shown in Table 3. The similarity between synthetic FA and real FA was nearly identical to that between grayscale color fundus images and real FA. This is unsurprising as most structures in FA are visible and similar in color fundus. The difference between the two modalities (FA and color fundus) will be important in NPA assessment, but such minor differences do not affect the image similarity metrics.

**Table 3:** Similarity metrics of the synthetic FA and color fundus to the FA.

	SSIM	LPIPS
Synthetic FA	0.507	0.091
Grayscale	0.526	0.087

Structural Similarity Index (SSIM) and Learned Perceptual Image Patch Similarity (LPIPS) between the true FA images and synthetic FA or grayscale color fundus images are shown. The higher SSIM and lower LPIPS indicate more similarity.

### Segmentation

**Figure 3:**
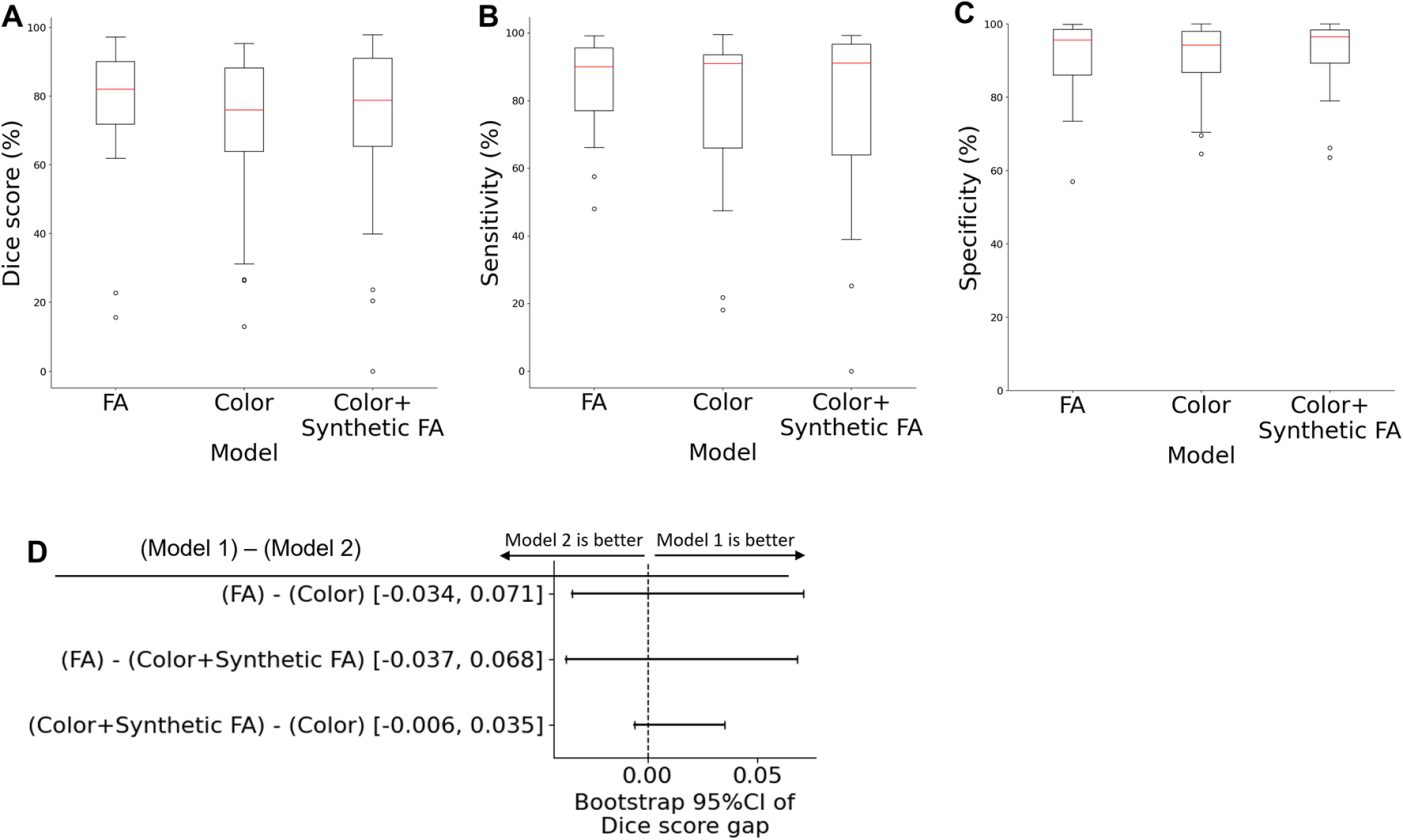
Accuracy of NPA prediction with different input sources. The red line indicates median, and the whiskers show 1.5 times IQR. (**A**–**C**) Dice score, sensitivity, and specificity of models with different input. (**D**) The bootstrap 95% confidence intervals of the sample-wise gap in Dice score between the two models. No statistical significance was observed in any pairs.

The FA model achieved the best accuracy with a median Dice score of 82.0%; however, the color fundus model also demonstrated comparable performance (Fig. 3A–C). The color fundus + synthetic FA model performed slightly better than the color fundus model but did not outperform the FA model. While not statistically significant, the confidence intervals in Fig. 3D suggest that the FA model likely performed the best, followed by the color fundus + synthetic FA model, and lastly the color fundus model.

The FA model yielded more stable predictions than other models. Although the median Dice score and sensitivity were similar to other models, their interquartile ranges (IQR) were narrower. Additionally, the Dice score was greater than 60% for all samples except for two, and the sensitivity was greater than 50% for all samples. In contrast, the other models, namely color fundus and color fundus + synthetic FA models, exhibited wider IQRs, lower minimum Dice scores, and lower sensitivities.

Even though our models generally performed well with acceptable Dice scores, wide IQR and outliers suggest there exists a high variability in performance. We extracted samples with low (< 40%) Dice scores in at least one model (Supplementary Fig. 1, Supplementary Table 1).

### Uncertainty estimation

**Figure 4:**
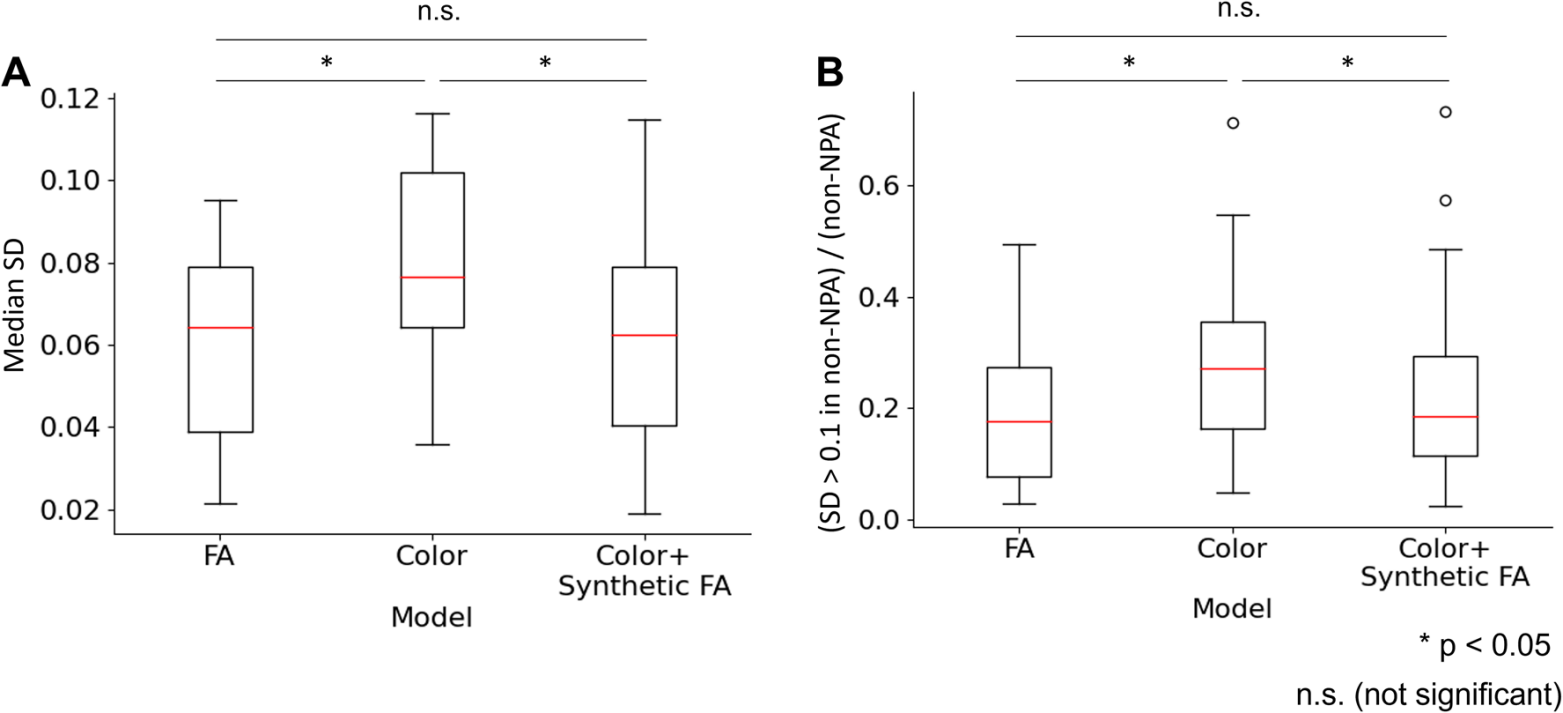
Monte Carlo dropout uncertainty. Standard deviation (SD) was acquired from 100 predictions using Monte Carlo dropout. (**A**) Median SD in each image, measuring the degree of uncertainty in general. (**B**) Ratio of pixels with SD > 0.1 to non-NPA pixels, representing the uncertainty outside true NPAs. For both measures, the uncertainty in the color fundus model was larger than the others with statistical significance.

The Monte Carlo dropout uncertainty was significantly higher in the color fundus model than in the other two models (Fig. 4). The difference was statistically significant for the median standard deviation (SD) and the proportion of the area with SD > 0.1 in non-NPA pixels. Although the FA model demonstrated lower uncertainty compared to the color fundus + synthetic FA model, the difference was not statistically significant.

### Analysis of individual samples

Examination of individual samples revealed that the influence of GAN-generated FA on segmentation accuracy varied across the dataset. Specifically, the addition of synthetic FA either improved, worsened, or had no impact on the Dice score, depending on the samples.

**Figure 5:**
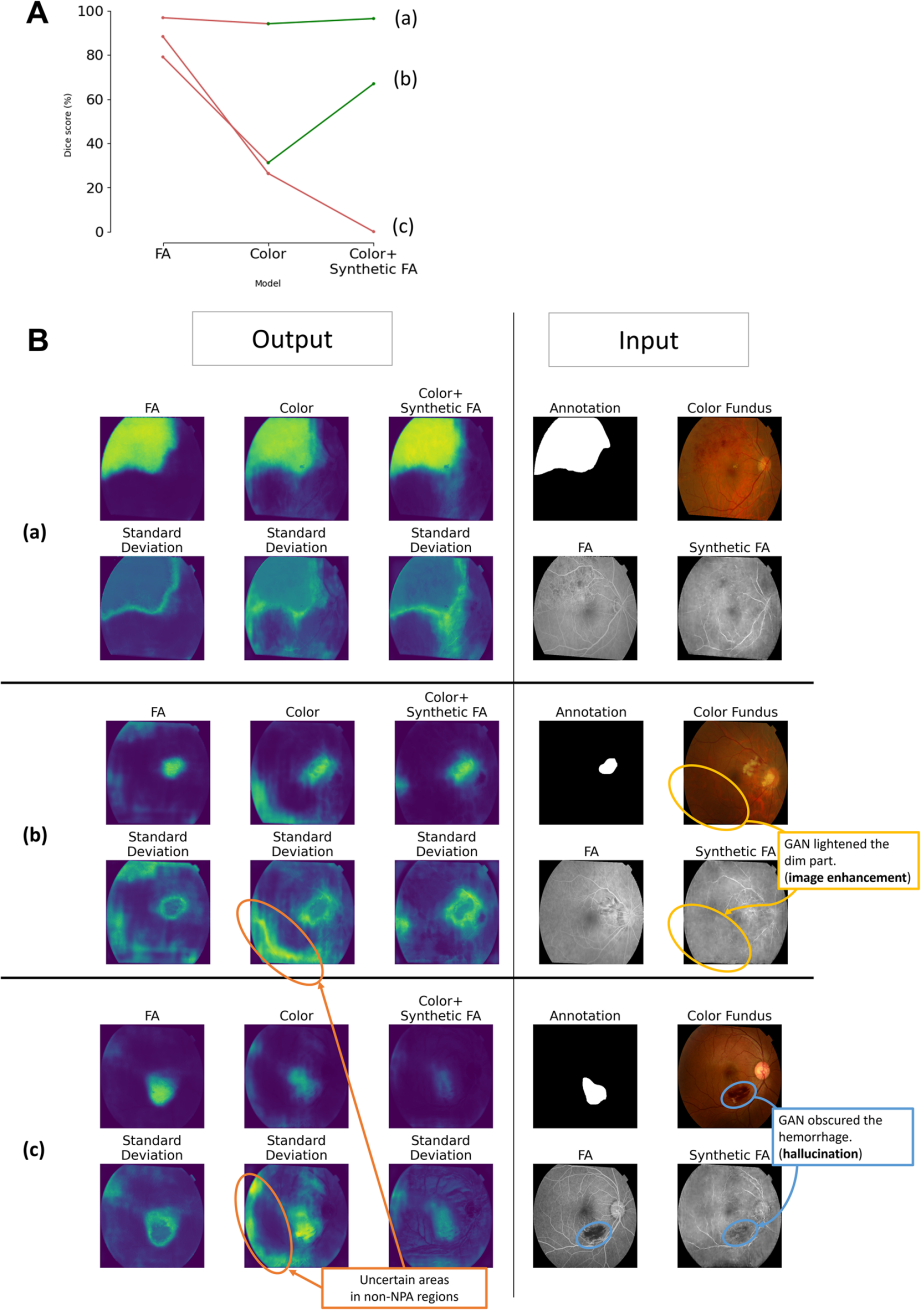
Representative samples of prediction and uncertainty. (**A**) Dice scores of three representative samples on different models. (**B**) Input and output of each sample. Samples (**a**)–(**c**) are correspondent in (**A**) and (**B**). (**a**) All models yielded accurate predictions. (**b**) The color fundus model lagged in accuracy compared to the FA model due to shadowing, which was, however, mitigated by synthetic FA. (**c**) The use of GAN reduced accuracy by obfuscating key details in the color fundus images. Meanwhile, the color fundus model had high-uncertainty area in non-NPA regions.

Figure 5 showcases three representative cases. Synthetic FA lightened the shadowing and enhanced image clarity (highlighted in the yellow circle). As a result, the model using synthetic FA displayed fewer uncertain areas in non-NPA regions (orange circle). This observation aligns with the earlier noted reduction in areas with SD > 0.1 in non-NPA regions by the color fundus + synthetic FA model. However, synthetic FA had the downside of obscuring abnormalities such as hemorrhages, leading to inaccurate predictions (blue circle).

## Discussion

In this research, we compared the FA model and two non-FA models in terms of accuracy and uncertainty. We also examined the clinical utility of synthetic FA generated from color fundus images. As a result, the FA model achieved the best accuracy, while the other two models also attained comparable accuracy. As for uncertainty, the FA model yielded the most stable prediction, while the color fundus model showed the highest Monte Carlo uncertainty.

Despite the comparable accuracy, the prediction from the non-FA models was unstable compared to the FA model. This is likely because some color fundus images lacked visible abnormalities such as hemorrhages. In other words, the accuracy of NPA prediction using color fundus was inconsistent; when lesions are visible in both color and FA images, the color fundus model can perform comparably to the FA model; however, when lesions are only visible in FA, the color fundus model performs worse than FA. Furthermore, the volatile imaging quality of the color fundus images might also have destabilized the color fundus model. Compared to FA, color fundus images are more vulnerable to image artifacts such as the angle of the camera and the direction of the lighting (e.g., shadows). These artifacts can lower the Dice score by deteriorating the image quality.

Visual inspection of these error samples revealed two common error scenarios. The first one is false-positive arising from bleeding or unclear regions (Samples A and B in Supplementary Fig. 1). The root cause of this error may be our conservative annotation policy that encourages erring on the safe side. Including more edge cases in the dataset and refining the annotation criteria to adapt to those cases would mitigate this issue. The other scenario is predictions with low confidence (Samples C-F in Supplementary Fig. 1). As Dice scores are calculated for binarized output with a threshold of 0.5, weak prediction vanishes even when models successfully locate NPAs, leading to low Dice scores. Adding more samples to the training set could enable models to make bolder predictions, particularly for typical cases. Also, heuristic calibration of the threshold that balances the risk of false-positive and false-negative might be helpful when applying these models to clinical settings in the future. Nevertheless, even with these issues, none of the error samples were so far off the mark from true NPAs that even an ophthalmologist would be at a loss to judge.

The impact of synthetic FA on accuracy was mixed; it led to both increases and decreases in Dice scores, depending on the samples. Meanwhile, synthetic FA consistently reduced Monte Carlo uncertainty, likely due to GAN’s capability of image enhancement. As previously mentioned, some color fundus images suffer from quality issues, leading to greater uncertainty. However, GANs can improve image quality. In fact, GANs are commonly used in image enhancement tasks such as noise reduction and super-resolution^15–19^. In this study, our GAN model presumably acquired image enhancement capability because the target images (FA) had better image quality than the source images (color fundus).

By using synthetic FA, we could reduce the misleading uncertainty estimates. This improvement is clinically beneficial. Uncertainty can help clinicians identify areas requiring further examination for abnormalities. However, "false alarms" of uncertainty estimates can mislead clinicians into investigating completely normal areas, thereby wasting their time. By using synthetic FA for NPA prediction, such false alarms can be decreased, and therefore the uncertainty estimates will be more reliable and helpful.

While the integration of generative AI into medical practice offers promising advancements, it is not without risks. One significant concern is the phenomenon of 'hallucination', where the AI generates non-existent information, potentially leading to misdiagnosis^20,21^. For example, there is a risk that AI models might obscure critical abnormalities, thereby preventing patients from receiving timely and appropriate care. This raises profound ethical, legal, and safety issues. An instance of this was observed in our study, specifically illustrated in Fig. 5Bc, where the model occasionally failed to highlight abnormalities. This likely occurred because the GAN model, despite being trained on images containing NPAs, was predominantly exposed to normal parts of FA, inadvertently biasing it towards generating normal results. Before deploying AI in clinical settings, it is crucial to rigorously evaluate its benefits against potential harms. Medical practitioners should be thoroughly educated about the capabilities and limitations of AI technologies. Additionally, AI-generated diagnoses or recommendations should undergo rigorous review by clinicians to mitigate risks of misdiagnosis. The ethical, legal, and social implications of employing generative AI in medicine remain significant, under-explored areas that require a deeper understanding of AI's capabilities and limitations. We hope that our research contributes valuable insights into this ongoing discourse.

Although our results suggest the limited effect of synthetic data on accuracy, some studies have reported that GAN-generated images could improve prediction performance in medical image tasks such as contrast-enhanced CT synthesis and other medical fields such as pathology^22,23^. Collectively, it is suggested that the effectiveness of GANs is task-dependent. In general, GANs cannot acquire additional information from patients; they can only refine existing features within the existing images. There should be a substantial limitation in that it cannot detect what is not there. Therefore, theoretically, using GAN-generated images for downstream tasks would only be beneficial when downstream models fail to extract features effectively.

Our analysis was limited due to the small size of the dataset, which could have affected the accuracy of the segmentation model and GAN model. Collecting additional data would not only augment the dataset’s volume but also enhance its quality. This enhancement arises from the ability to stratify the dataset, thereby facilitating a more homogeneous dataset across various stages in the course of RVO (e.g., first visit and follow-up). Furthermore, due to computational resource constraints, we had to evaluate the models using the hold-out method, which is less robust and generalizable in small datasets than the cross-validation method. Therefore, more extensive research is needed to determine the utility of synthetic data in medical image AI. Future work should aim to develop a more stable NPA segmentation model that performs well even when abnormalities are subtle or not readily apparent on color fundus images.

Furthermore, the present research examined the potential utility of synthetic FA images in the limited context of segmenting NPAs in RVO patients. However, FA has broader clinical utility beyond NPA detection, and it is frequently used in the diagnosis of a variety of retinal diseases including diabetic retinopathy, age-related macular degeneration, and more. We only shed light on one of them, and further research is needed to examine the utility of synthetic FA images in the broader clinical context.

In conclusion, the deep learning models can predict NPAs solely from color fundus images with acceptable accuracy. This result is prospective towards the aim of providing BRVO patients with safe and accessible examinations. However, at this point, NPA prediction relying solely on color fundus images can lead to missed lesions, given its instability. Further research is needed to overcome this challenge. The unstable performance can be attributed to two factors. First, the color fundus model performs comparably only when there are visible lesions of NPA in the color fundus images. When an input image completely lacks indicative features, the model performance deteriorates. Second, the quality of color fundus images is more likely to be impaired than FA due to image artifacts such as shadowing. The primary contribution of the GAN-generated FA is its image enhancement effect such as noise reduction and brightness adjustment. Although the improvement in accuracy is subtle, GAN-generated FA lowers “false alarm” in Monte Carlo dropout uncertainty estimates and thereby enhances their clinical utility as an indicator for requiring doctors’ further inspection of a specific part in a fundus image.

## Materials and methods

### Ethical statement

The study was conducted in accordance with relevant guidelines and regulations including the Declaration of Helsinki and was approved by the institutional review board of the Keio University School of Medicine (approval no. 20170049). Due to the retrospective observational nature of this study, the informed consent was obtained through an opt-out approach from all participants. Identifying information was anonymized prior to analysis. The study involved no interventions in humans or animals.

### Dataset

We retrospectively collected 403 sets of color fundus and FA images from 319 BRVO patients at the Keio University School of Medicine, between July 28, 2011, and August 26, 2019. We analyzed photographs taken across different times without distinction of baseline or follow-up; however, we prioritized images without hemorrhage obstruction when multiple images were available for a single patient. The annotations for the NPAs were performed by three licensed ophthalmologists, using both color fundus and FA images for reference. They also aligned the color and FA images by an affine transformation. The low-quality samples, on which doctors could not make a diagnosis, were excluded from the dataset. The annotators were encouraged to err on the side of false positives rather than miss potential lesions in case of bleeding or unclear boundaries. After the annotations were completed by three independent ophthalmologists, we generated ground truth using the union set of the NPA maps by the three annotators.

The dataset was then divided into training (330 images), validation (38 images), and test (35 images) subsets. Each subset had specific roles in segmentation and synthetic FA generation. In the segmentation task, the roles of each dataset were straightforward: the training set for model training, the validation set for monitoring generalization performance, and the test set for final evaluation. To avoid overfitting and ensure unbiased evaluation, the test set was never used except for the final evaluation. For the training of the synthetic FA generation model, we used the validation set to minimize potential data leakage between the segmentation and generation models, ensuring an unbiased evaluation. Using the same dataset for both segmentation and generation models could lead to data leakage, and the segmentation model would be able to exploit this leakage. The segmentation model using synthetic FA would have indirect access to FA during training, which however would not occur in test or real-world use.

### FA synthesis

To generate synthetic FA from the color fundus, we utilized generative adversarial networks (GANs)^24,25^. This technique is widely used in image generation across various fields, including medicine^26–29^. Specifically, we used Fundus2Angio architecture, which was designed for color-to-FA translation^10^. For more details on the model, readers are encouraged to refer to the original paper. The model architecture and hyperparameters used in the present research are the same as ones of the original authors' implementation available at their GitHub repository.

The quality of the generated synthetic FA images was measured by two metrics: Structural Similarity Index (SSIM) and Learned Perceptual Image Patch Similarity (LPIPS)^30,31^. Both measures are generally used to quantify the similarity between two images. SSIM focuses on the luminance, contrast, and structure of two images, while LPIPS leverages deep learning to capture complex visual similarities, better aligning with human perception. We also calculated these metrics for the grayscale images of the color fundus for comparison.

### Segmentation

For the segmentation of NPAs, we used U-Net with Monte Carlo dropout^32,33^. U-Net is a widely used medical image segmentation model, and Monte Carlo dropout is an uncertainty estimation method for deep learning models. By combining these methods, we can analyze both the prediction accuracy and uncertainty estimates. We opted for U-Net due to its straightforward architecture. Our previous research indicated that a deep learning model with simple architecture produces more informative uncertainty estimation^34^.

The segmentation model was trained with different input types: (1) FA model, (2) color fundus model, and (3) color + synthesized FA model. The models were trained with a batch size of 4, the Adam optimizer, an initial learning rate of 0.0004, and the cross-entropy loss function.

### Evaluation

Using the test subset, we evaluated the accuracy of the segmentation models by the Dice coefficient score and sensitivity. Sensitivity is the ratio of true-positive pixels to the total NPA pixels in an image. Since we cannot assume the Gaussian distribution on the Dice score, the Wilcoxon signed-rank test was used to test the difference in prediction performance. The confidence intervals for differences in Dice scores were calculated using the bootstrap method.

Additionally, we calculated the standard deviation (SD) from 100 Monte Carlo dropout predictions for uncertainty estimation. To compare the nature of each model in terms of uncertainty, the median SD and the area fraction with SD > 0.1 were quantified and compared using the Wilcoxon signed-rank test. Furthermore, we performed individual-level comparisons to assess the impact of the absence of FA or the inclusion of synthesized FA on a case-by-case basis. For multiple testing correction, the Bonferroni method was applied.

### Supplementary Information

Supplementary Information.

## Data Availability

The dataset is not publicly available due to their containing information that could compromise the privacy of research participants but may be available from the corresponding author upon reasonable request.

## Abbreviations

AI: Artificial intelligence
BAVO: Branch retinal vein occlusion
NPA: Non-perfusion area
FA: Fluorescein angiography
GAN: Generative adversarial network
MC: Monte carlo
SSIM: Structural similarity index
LPIPS: Learned perceptual image patch similarity
