# Supplementary material for: Deep learning segmentation of non-perfusion area from color fundus images and AI-generated fluorescein angiography
Source: Sci Rep. 2024 May 11;14:10801. doi: 10.1038/s41598-024-61561-x (PMC11088618; doi:10.1038/s41598-024-61561-x)
Supplement: Supplementary file 1 — Supplementary Information. [file 41598_2024_61561_MOESM1_ESM.pdf]

Supplementary Information

Deep Learning Segmentation of Non-perfusion Area from Color Fundus Images and AI-generated Fluorescein Angiography

Kanato Masayoshi[1, *] Yusaku Katada[1,2, *], Nobuhiro Ozawa[1,2], Mari Ibuki[1,2], Kazuno Negishi[2], Toshihide Kurihara[1,2]

[*]: These authors contributed equally to this work.

[1]Laboratory of Photobiology, Keio University School of Medicine, Shinanomachi, Shinjuku-ku, Tokyo, Japan.

[2]Department of Ophthalmology, Keio University School of Medicine, Shinanomachi, Shinjuku-ku, Tokyo, Japan.

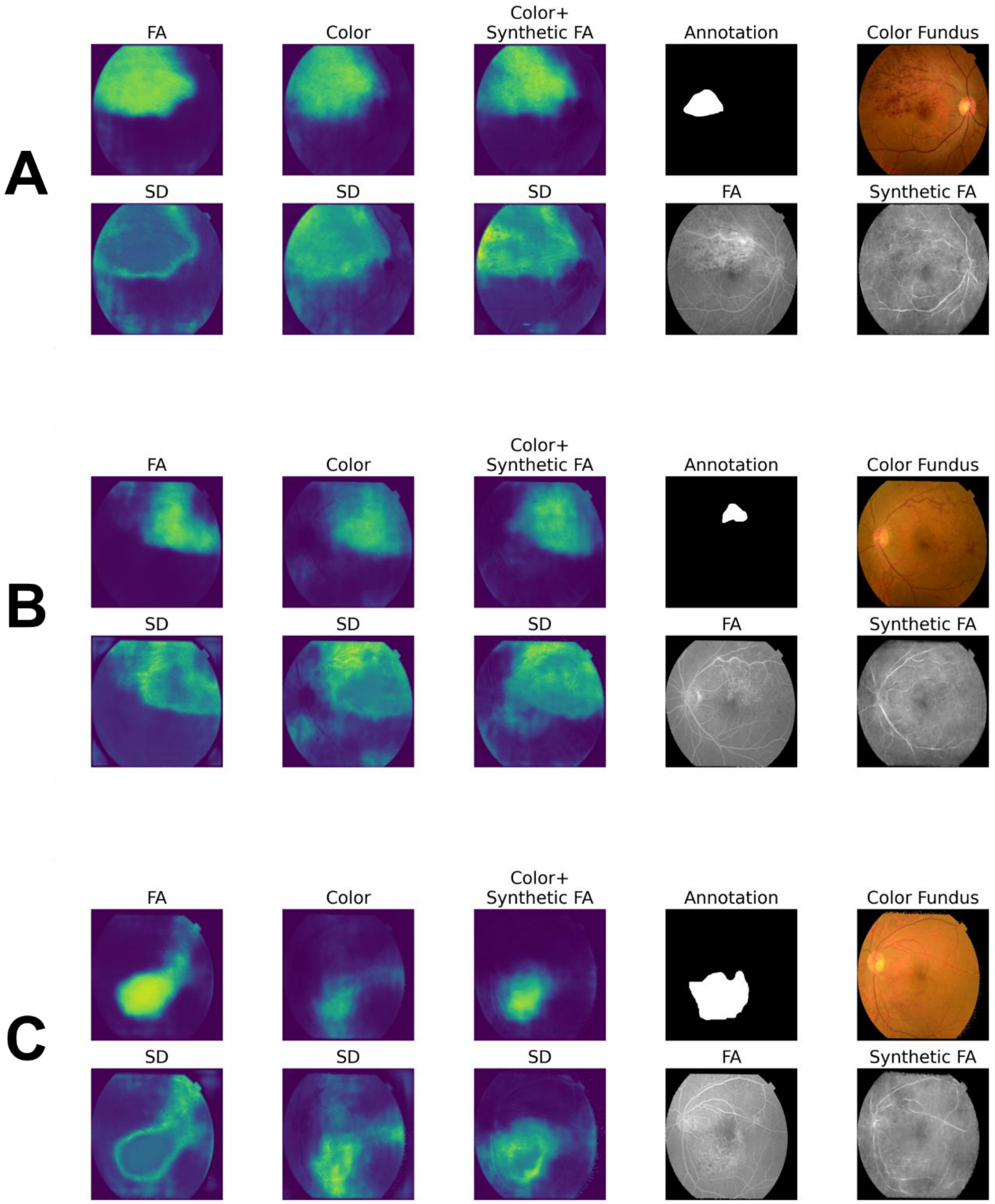

Supplementary Figure 1 (continues next page)

D

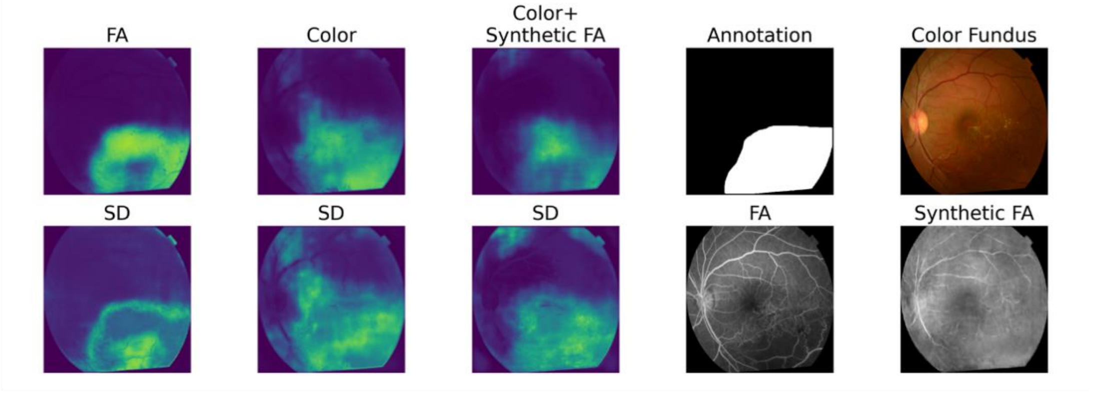

E

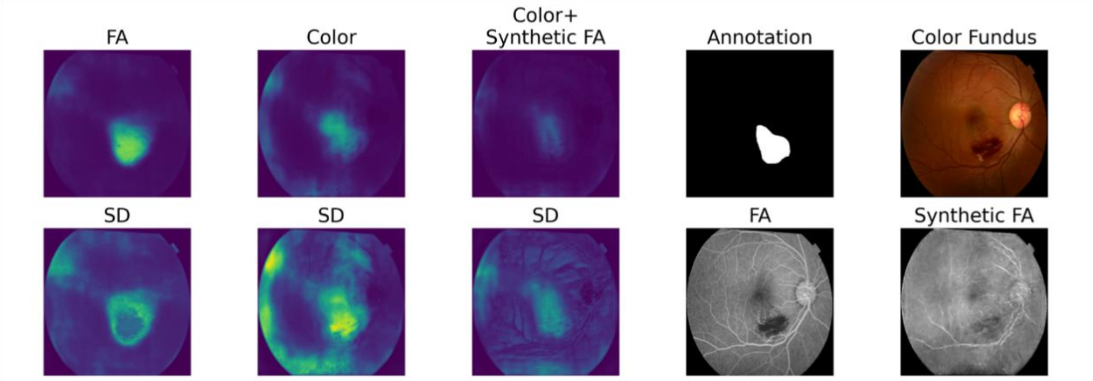

F

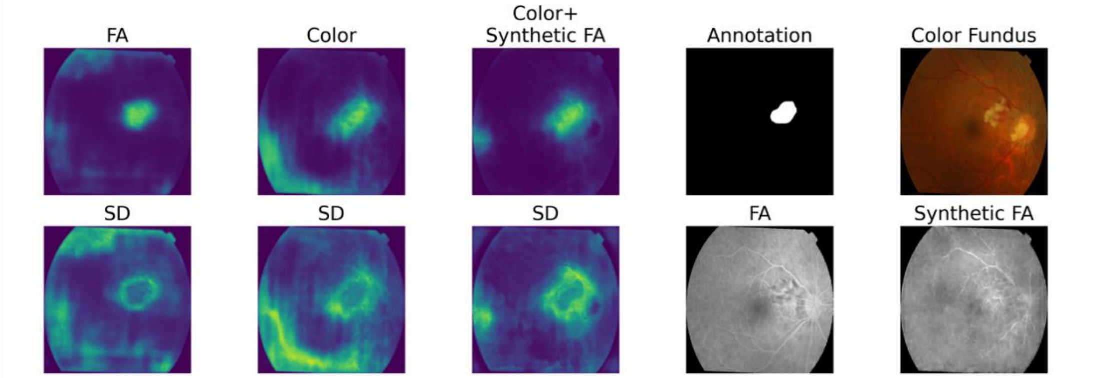

Supplementary Figure 1 (continued) Error samples with low Dice scores.

We present six samples from the test dataset that demonstrated Dice scores below 40% in at least one of the models. It is important to note that the prediction maps display the models' raw output. Dice scores were calculated after binarizing each output value using a threshold of 0.5. "SD" denotes standard deviation, employed here as an uncertainty metric.

Supplementary Table 1 Dice scores of error samples

Sample	Dice Score (%)		
	FA	Color	Color+Synthetic FA
A	22.8	26.6	20.4
B	15.7	12.9	23.7
C	82.0	35.9	69.0
D	83.1	76.0	39.8
E	88.4	26.4	0.0
F	79.1	31.2	67.0

Dice scores below 40% are highlighted red.
